# Management and outcomes of pregnant ICU patients with severe COVID-19 pneumonia in Qatar: A retrospective cohort study

**DOI:** 10.5339/qmj.2023.24

**Published:** 2023-12-12

**Authors:** Hayat Elfil, Mogahed Ismail Hassan Hussein, Layla J. M. Kily, Sohel M. G. Ahmed, Mohammed Janish, Salwa M. Abuyaqoub, Huda A. Saleh, Marcus D. Lance

**Affiliations:** ^1^Department of Anesthesiology, ICU and Perioperative Medicine, Women’s Wellness and Research Centre, Doha, Qatar; ^2^Department of Anesthesiology, ICU and Perioperative Medicine, Hamad General Hospital, Doha, Qatar Email: Helfil@hamad.qa ORCID iD: 0000-0002-8210-0640; ^3^Department of Anesthesiology, ICU and Perioperative Medicine, Research, Hamad Medical Corporation, Doha, Qatar; ^4^Department of Obstetrics and Gynaecology, Women’s Wellness and Research Centre, Doha, Qatar; ^5^Department of Anesthesiology, ICU and Perioperative Medicine, Aga Khan University Medical College, Nairobi, Kenya

**Keywords:** COVID-19, intensive care unit, non-invasive ventilation, intermittent positive pressure ventilation, high-flow nasal cannula, pregnancy, Qatar

## Abstract

Introduction: Pregnant women are considered a high-risk group for COVID-19 due to their increased vulnerability to viral infections. The impact of COVID-19 on pregnant women is not well understood, and there is a need for data on managing severe COVID-19 in pregnant patients. This retrospective descriptive cohort study described the characteristics, hospital stay, interventions, and outcomes of pregnant patients admitted to the intensive care units (ICUs) with severe COVID-19 pneumonia in Qatar.

Methods: Data were collected from medical records and chart reviews of pregnant women admitted to Hamad Medical Corporation (HMC) with COVID-19 pneumonia from March 01, 2020, to July 31, 2021. The inclusion criteria encompassed pregnant women with a positive polymerase chain reaction (PCR) antigen test or radiological changes at admission, requiring respiratory support, and hospitalized for more than 24 hours.

Results: A total of 43 pregnant women were included in this study. Most patients were admitted during the first wave of the pandemic, with a median gestational age of 212 days [interquartile range 178–242 days] at presentation. The most common respiratory support methods were high-flow nasal cannula, non-invasive positive pressure ventilation, and invasive positive pressure ventilation. Convalescent plasma therapy was administered to 58% of patients, and tocilizumab was used in 28%. Renal replacement therapy was required by 4.6% of patients and 7% required extracorporeal membrane oxygenation.

Conclusion: This study provides valuable insights into the impact of COVID-19 on pregnant patients admitted to the ICUs in Qatar. The results suggest that pregnant patients with COVID-19 pneumonia require close monitoring and appropriate interventions to minimize adverse outcomes for both mother and fetus. The data may contribute to future guidelines and management strategies for severe COVID-19 in pregnant patients.

## Abbreviations

BiPAP: bilevel positive airway pressure, BMI: body mass index, CPAP: continuous positive airway pressure, CRRT: continuous renal replacement therapy, ECMO: extracorporeal membrane oxygenation, HFNC: high-flow nasal cannula, ICU: intensive care unit, IPPV: intermittent positive pressure ventilation, NICU: neonatal intensive care unit, NIV: non-invasive ventilation, RRT: renal replacement therapy, SARS-CoV-2: severe acute respiratory syndrome coronavirus 2, SLED: sustained low-efficiency dialysis.

## Introduction

COVID-19, caused by the severe acute respiratory syndrome coronavirus 2 (SARS-CoV-2), presents as a respiratory illness affecting individuals differently based on age, gender, pre-existing health conditions, obesity, and pregnancy.^1^ Women are generally less affected by SARS-CoV-2 compared to men,^1^ but pregnant women are considered a high-risk group due to their increased vulnerability to viral infections.^2^ Previous pandemics, such as in 1918, 1957, and 2009 flu outbreaks, have shown high mortality rates among reproductive-age women, including pregnant women.^3−5^

Additionally, pregnancy can lead to conditions such as gestational diabetes and hypertensive disorders, which are known risk factors for severe COVID-19.^6^ It was anticipated that pregnant women might suffer more from COVID-19 compared to non-pregnant women of a similar age;^7^ however, current studies on COVID-19’s impact on pregnancy are conflicting, and it is unclear if pregnancy is a protective or an additional risk in the context of the illness.^8^

Healthcare providers must monitor and manage COVID-19 in pregnant women to minimize the risk of adverse outcomes for both the mother and the fetus. This includes providing appropriate treatment, education, and support to help reduce stress and anxiety related to COVID-19.

In Qatar, a recent study revealed that nearly 70% of pregnant women with COVID-19 infection reported at least one disease-related symptom, such as cough, fever, fatigue, or myalgia. Furthermore, this study reported a higher proportion of Qatari women, older age, grand multiparous, obesity, pre-existing diabetes mellitus (DM), and gestational diabetes mellitus (GDM) compared to expected national figures.^9^

This retrospective descriptive cohort study aims to describe the characteristics of pregnant patients admitted to the intensive care unit (ICU) with severe COVID-19 infection in Qatar during the pandemic’s first and second waves (March 01, 2020, to July 31, 2021). This study will provide valuable insights into the impact of COVID-19 on pregnant patients admitted to the ICU at Hamad Medical Corporation (HMC). The results of this study might support future guidelines and strategies for managing severe COVID-19 infection in pregnant patients and contribute to our understanding of the disease and its effects on this vulnerable population.

## Methodology

This retrospective descriptive cohort study aims to describe the characteristics, hospital stay, interventions, and outcomes of pregnant patients admitted to the ICU with severe COVID-19 pneumonia in Qatar. The primary aim of this study was to identify the sub-cohort requiring ventilation and/or oxygenation support. This study provides insight into the impact of COVID-19 pneumonia on pregnant women and the management strategies used to mitigate its effects.

This retrospective chart-based cohort study analyzed the characteristics of pregnant women admitted with COVID-19 pneumonia in HMC. The data were collected from medical records and chart reviews from our electronic health system (Cerner-Oracle). The inclusion criteria encompassed pregnant women admitted with COVID-19 pneumonia diagnosed with a positive polymerase chain reaction (PCR) antigen test or radiological changes at admission. These women also required respiratory support in the form of non-invasive ventilation (NIV), continuous positive airway pressure (CPAP), bilevel positive airway pressure (BiPAP), high-flow nasal cannula (HFNC), or any form of intermittent positive pressure ventilation (IPPV). The exclusion criteria included patients who did not require hospitalization or were discharged within 24 hours.

A total enumeration sampling was used, including all pregnant women hospitalized with a primary diagnosis of COVID-19 pneumonia requiring assisted ventilation during the study period of March 01, 2020, to July 31, 2021 (covering both the first and second waves of the pandemic in Qatar).^10,11^

The data were collected in Excel (Microsoft Excel) spreadsheets and analyzed using IBM SPSS 22 (IBM, Chicago, IL, USA). The HMC Institutional Review Board (IRB), Medical Research Centre (MRC), under Protocol No MRC-01-21-790, waived the need for ethical approval.

## Results

The results of this retrospective descriptive study show the characteristics of 43 pregnant women who were admitted to the ICU with severe COVID-19 pneumonia and received either NIV or IPPV (Table 1). Out of this cohort, outcome data of three pregnancies was missing. Hence, they were excluded from the analysis of patients’ characteristics, including their body mass index (BMI), age, nationality, pregnancy history, and pre-existing health conditions (Table 1). As expected, our sample was multinational and representative of the population of Qatar. The mean age of the cohort was 32.6 ± 4.88 years. Twenty-two patients (51.2%) were obese, with a BMI of 30 kg/m^2^ or more. Only one patient had essential hypertension (2.3%). The median gestational age at presentation was 212 days [interquartile range 178–242 days]. The patients had a range of gravidity from 1 to 10, with the majority being gravida 2 (23.3%). Thirty-two patients were admitted during the first wave of the pandemic. None of these patients was vaccinated, as most presented before the COVID-19 vaccine was developed and available in Qatar. Chest X-rays were unavailable for two patients, but among those who had X-rays, the majority showed primarily bilateral infiltrates (44%).

Twenty-nine out of the forty cases of COVID-19 pneumonia presented during the third trimester of pregnancy. The most common respiratory support methods utilized were HFNC in 67.5% of cases, NIV in 47.5%, and IPPV in only 40%. Only 14% required vasopressors, with five patients receiving noradrenaline and one receiving adrenaline infusions. Convalescent plasma therapy was given to 58% of patients (25 patients), and tocilizumab was used in 28% of patients (12 patients), guided by interleukin levels.

Three patients (7.5%) required extracorporeal membrane oxygenation (ECMO), of which two patients required renal replacement therapy (RRT). The patient who received sustained low efficiency dialysis (SLED) was in the ECMO group and had intrauterine fetal demise. The third ECMO case did not require RRT. Two had a preterm pregnancy outcome and were delivered via lower segment caesarean section (LSCS), and both neonates required admission to the neonatal intensive care unit (NICU).

**Figure 1.**
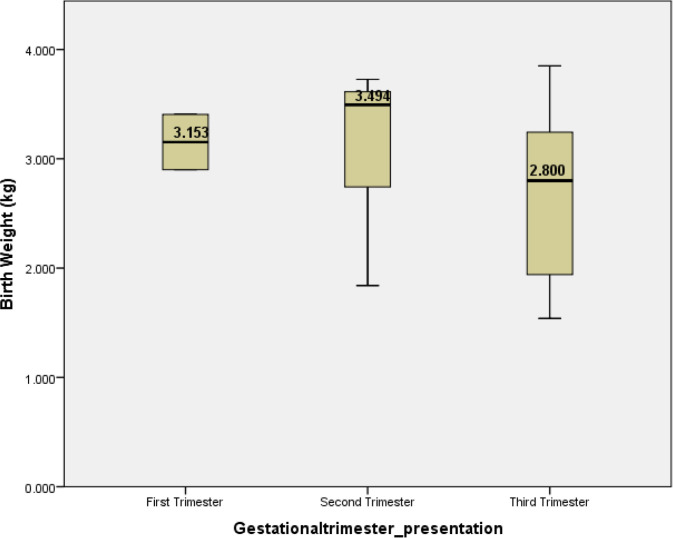
The graph illustrates the relationship between gestational age at presentation and birth weight. There is an overlap in the confidence intervals for all three groups, indicating no significant difference in birth weight among infants born to mothers who presented at different gestational ages.

**Figure 2.**
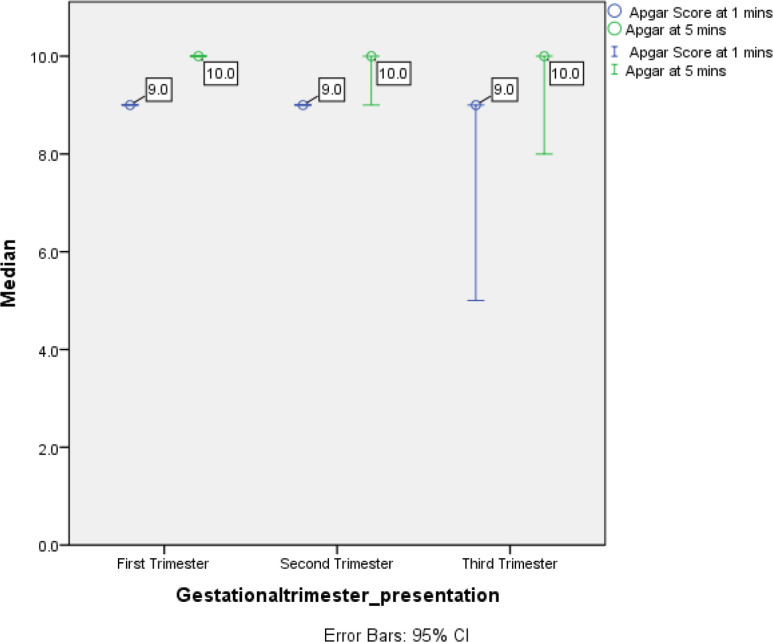
The graph illustrates the relationship between gestational age at presentation and Apgar scores at 1 minute (blue line) and 5 minutes (green line) for a group of individuals. The graph suggests some overlap exists between the Apgar scores of neonates born to mothers who presented at different gestational ages.

Regarding the pregnancy outcome, mean fetal birth weights were 3.153 kg, 3.494 kg, and 2.8 kg in women infected with COVID-19 in their first, second, and third trimesters, respectively (Figure 1). Apgar scores at 1 minute were 9 and 10 at the 5th minute for all born fetuses (Figure 2). Applying the student t-test, no significant association was found between the type of respiratory support used and pregnancy outcomes, as summarized in Table 2.

Of the remaining 40 patients, 39 had live fetuses, with 22 born full-term and 17 preterm. One case of intrauterine fetal demise was recorded. Most deliveries (67.5%), i.e., 27 out of the 40 pregnancies, were done via lower segment caesarean section, with 13 patients having normal vaginal deliveries, 8 of whom received epidural anesthesia.

## Discussion

This study demonstrates that in a cohort of pregnant women at different gestational ages presenting with respiratory distress due to COVID-19 pneumonia, the outcomes for both the mother and the fetus are comparable to those of non-infected pregnant women. Similar results are seen in other studies with various sample sizes, which reported favorable results in pregnant women.^12−17^ Infection rates among pregnant women in Wuhan, China, were similar to the general population, with no maternal deaths reported among 11,078 COVID-19 cases.^18^ In Genoa, Italy, 6 out of 325 asymptomatic pregnant women tested positive for COVID-19, but none developed symptoms, and the newborns were not infected.^14^ Adverse COVID-19-associated outcomes among pregnant women seem to be linked to underlying conditions such as advanced age, obesity, hypertension, and pre-existing diabetes rather than pregnancy.^19^ Hospitalized pregnant women with COVID-19 were likelier to be overweight and have medical conditions such as asthma and hypertension.^20^ Obesity was also a risk factor for severe COVID-19 in pregnant women in Italy.^21^ Likely, pregnant women without these underlying conditions are not disproportionately impacted by SARS-CoV-2.^8^

In a retrospective-controlled study conducted in Qatar, Abdalla et al. found no significant differences in neonatal outcomes between pregnant women with COVID-19 and those without the infection.^10^ The study included 64 mothers in each group and found that most neonates in both groups had average birth weights and Apgar scores and were discharged from the hospital in good health. There were slightly higher rates of emergency caesarean deliveries and assisted vaginal deliveries in the COVID-19 group, but this did not result in adverse neonatal outcomes. Two neonates in the COVID-19 group tested positive for SARS-CoV-2, were admitted to the NICU, and discharged from the hospital in good health. The study suggests that COVID-19 infection during pregnancy does not impact neonatal outcomes significantly.^19^

In line with the case–control study conducted in Qatar,^19^ which found no significant differences in neonatal outcomes between pregnant women with and without COVID-19 pneumonia, our retrospective descriptive study did not observe any significant association between gestational age at presentation with COVID-19 pneumonia and the modality of the respiratory support used. Furthermore, our study found that most neonates in our cohort had a term pregnancy outcome and did not require NICU admission. Additionally, respiratory support, including HFNC, NIV, and IPPV, did not appear to have a significant association with pregnancy outcomes in our study.

One study found that most pregnant women hospitalized with SARS-CoV-2 infection were in the late second or third trimester of pregnancy. The outcomes for these patients were primarily positive, with a low incidence of vertical virus transmission from mother to infant.^21^ Both studies found that most pregnant women with COVID-19 infection were in the late second or third trimester. Overall, both studies suggest that COVID-19 infection during pregnancy does not impact neonatal outcomes significantly.

Another study conducted in a general population of COVID-19 patients admitted to the ICU identified various risk factors associated with mortality.^22^ The study found that advanced age, respiratory failure, mechanical ventilation, and elevated procalcitonin levels were significantly associated with higher mortality rates. These findings highlight the importance of considering these factors when assessing the severity and prognosis of COVID-19 patients.

## Limitations

This study had several limitations that should be considered when interpreting the results. Firstly, the study design was retrospective, which may have introduced bias due to the reliance on existing data. This study only included a single cohort, which may limit the generalizability of the findings to other populations or settings. Another study limitation was the exclusion of some patients due to missing data, which may have affected the sample size and potentially biased the results. Finally, this study did not control for confounding variables, such as demographic factors or comorbidities, which may have affected the study outcomes. While the findings of this study provide valuable insights, further research with more robust study designs is needed to confirm and expand upon these results.

## Conclusion

Our descriptive study on pregnant women with SARS-CoV-2 infection shows that most patients presented in the third trimester and received NIV, with no significant association between respiratory support and pregnancy outcomes. Even the need for invasive therapies such as RRT and ECMO did not occur frequently, and their effect was moderate on maternal and fetal outcomes.

## Conflict of Interests

All authors declare that they have no conflict of interest.

## Funding

No funding was received to conduct this study.

## Ethical Approval

The need for ethical approval was waived by the HMC Institutional Review Board (IRB), Medical Research Centre (MRC), under Protocol No. MRC-01-21-790.

## Figures and Tables

**Table 1.** Intensive care unit presentation characteristics of pregnant women at their first, second, and third trimesters (*n* = 2, *n* = 12, and *n* = 29, respectively).

Characteristics	First Trimester (*n* = 2)	Second Trimester (*n* = 12)	Third Trimester (*n* = 29)
Gestational Presentation (days)*	65 [19.79]	159.58 [22.33]	233.10 [23.11]
Gravidity†	5	2, 4	2, 3
Parity†	1	3	1
Age*	34 [7.07]	33.66 [5.41]	32.06 [4.64]
BMI*	36.12 [3.99]	33.73 [5.39]	31.37 [5.43]
Spoken Language‡			
Arabic Speaking	1 (50)	7 (58.3)	13 (44.8)
Non-Arabic speaking	1 (50)	5 (41.7)	15 (55.2)
COVID-19 Unvaccinated‡	2 (100)	12 (100)	29 (100)
COVID-19 Wave‡			
Wave 1st	2 (100)	9 (75)	21 (72.4)
Wave 2nd	0	3 (25)	8 (27.6)
Total hospital stay (days)*	13.5 [6.36]	17.83 [14.13]	21.89 [28.12]
Comorbities§			
Asthma	0 (0)	1 (2.28)	3 (2.76)
Obesity	1 (33.34)	5 (11.37)	16 (14.68)
Anemia	0 (0)	5 (11.37)	12 (11.01)
Gestational DM (T1 and T2)	1 (33.34)	5 (11.37)	11 (10.1)
Hypothyroidism	0 (0)	2 (4.55)	4 (3.67)
Primary HTN	1 (33.34)	0 (0)	0 (0)
PET	0 (0)	0 (0)	1 (0.92)
Eclampsia	0 (0)	0 (0)	2 (1.84)
A-Fib	0 (0)	1 (2.28)	0 (0)
Others	0 (0)	1 (2.28)	2 (1.84)
Symptoms§			
SOB	1 (11.12)	8 (19.05)	20 (19.61)
Malaise	1 (11.12)	0 (0)	2 (1.97)
Headache	0 (0)	1 (2.39)	4 (3.93)
Loss of smell/taste	0 (0)	1 (2.39)	0 (0)
Cough	1 (11.12)	12 (28.58)	26 (25.5)
Fever	2 (22.23)	7 (16.67)	18 (17.65)
Diarrhoea	0 (0)	1 (2.39)	3 (2.95)
Vomiting	0 (0)	0 (0)	3 (2.95)
Body pain	1 (11.12)	1 (2.39)	4 (3.93)
Others	3 (33.34)	11 (26.2)	23 (22.55)
Blood group‡			
A	0	0	1 (3.4)
A−	0	1 (8.3)	2 (6.9)
A+	0	2 (16.7)	5 (17.2)
AB+	1 (50)	3 (25)	3 (10.3)
B+	1 (50)	1 (8.3)	7 (24.1)
O−	0	1 (8.3)	7 (24.1)
O+	0	4 (33.3)	11 (37.9)
Blood parameters‡			
Hb	12.5 [0.77]	11.4 [0.72]	11.02 [1.27]
WBC	4.44 [1.91]	6.45 [3.23]	6.79 [1.89]
CRP	38 [25.4]	71.84 [66.23]	66.82 [39.66]
Creatinine	37 [22.62]	38.5 [6.45]	41 [10.851]
CT Value	18.22 [2.45]	20.01 [3.31]	24.62 [16.28]

^*^Mean [standard deviation], ^†^Mode, ^‡^Frequency (% within column), ^§^number (%) based on multiple responses.

COVID-19: coronavirus disease 2019, DM: diabetes mellitus, HTN: hypertension, PET: pre-eclamptic toxaemia, A-Fib: atrial fibrillation, SOB: shortness of breath, Hb: hemoglobin, WBC: white blood cells, CRP: C-reactive protein, CT Value: = cycle threshold value.

**Table 2.** A comparison of related parameters of pregnancy among three outcome groups: pre-term (*n* = 17), full-term (*n* = 22), and IUFD (*n* = 1).

Parameter(s)	Pre-Term (*n* = 17)	Full Term (*n* = 22)	IUFD (*n* =1)	Statisticsa *P*-value^d^
Gestational age (days)¶	222 [34]	211.5 [77.25]	0	*0.368^b^*	*0.713*
LOS ICU (days)¶	78 [10.5]	5 [6.5]	0	*1.281^b^*	*0.2*
Symptoms Status‡				*1.722^c^*	*0.423*
Symptomatic	17 (100)	20 (90.9)	1 (100)		
Asymptomatic	0	2 (9.1)	0		
Morbidity Status‡				*0.491^c^*	*0.782*
No morbid condition	2 (11.8)	4 (18.2)	0		
≥onemorbiditys	15 (98.2)	18 (81.8)	1 (100)		
BMI Classification‡				*3.532^c^*	*0.473*
Normal weight	1 (5.9)	2 (9.1)	0		
Overweight	4 (23.5)	5 (22.7)	0		
Obesity I	8 (47.1)	5 (22.7)	0		
Obesity II	2 (11.8)	7 (31.8)	0		
Obesity III	2 (11.8)	3 (13.36)	0		
Presentation age‡				*7.234^c^*	*0.124*
First Trimester	1 (5.9)	1 (4.5)	0		
Second Trimester	1 (5.9)	7 (31.8)	1 (100)		
Third trimester	15 (88.2)	14 (63.6)	0		
X-ray‡				*3.96^c^*	*0.41*
Bilateral infiltrates	9 (52.9)	6 (30)	1 (100)		
Consolidation	0	1 (5)	0		
Others	8 (47.1)	13 (65)	0		
Respiratory Support Factors				
HNFC‡				*0.733^c^*	*0.693*
No	6 (35.3)	6 (27.3)	0		
Yes	11 (64.7)	16 (72.7)	0		
Duration (hours)¶	1.35 [2]	1.5 [1.63]		*1.211^b^*	*0.251*
NIV‡				*2.257^c^*	*0.324*
No	7 (41.2)	13 (59.1)	0		
Yes	10 (58.8)	9 (40.9)	1 (100)		
Duration (hours)¶	0.65 [2.20]	1 [1.69]	0	*0.328^b^*	*0.743*
IPPV‡					
No	6 (35.3)	17 (77.3)	0		
Yes	11 (64.7)	5 (22.7)	1 (100)		
Duration (hours)¶	5 [6.52]	7 [17.73]	0	*0.914^c^*	*0.361*
Patient Management Factors					
VTE‡				*3.584^c^*	*0.465*
None	15 (88.2)	21 (95.5)	1 (100)		
PE	2 (11.8)	0	0		
DVT	0	1 (4.5)	0		
RRT#					
SLED	0	0	1 (100)		
CRRT	1 (100)	0	1 (100)		
ECMO‡				*14.563^c^*	***0.001***
No	15 (88.2)	22 (100)	0		
Yes	2 (11.8)	0	1 (100)		
Inotropes‡				*9.312^c^*	*0.052*
None	14 (82.4)	20 (90.9)	0		
Noradrenaline	3 (17.6)	1 (4.5)	1 (100)		
Adrenaline	0	1 (4.5)	0		
Convalescent plasma‡				*3.62^c^*	*0.164*
No	10 (58.8)	7 (31.8)	0		
Yes	7 (41.2)	15 (68.2)	1 (100)		
Tocilizumab‡				*4.112^c^*	*0.128*
No	11 (64.7)	18 (81.8)	0		
Yes	6 (35.3)	4 (18.2)	1 (100)		
Mode of delivery‡				*4.496^c^*	*0.106*
NVD	3 (17.6)	9 (40.9)	1 (100)		
LSCS	14 (82.4)	13 (59.1)	0		
Anesthesia Mode‡				*15.251^c^*	***0.002***
Spinal	4 (28.6)	11 (84.6)			
Epidural	0	1 (7.7)			
CSE	0	1 (7.7)			
GA	10 (71.4)	0			

^¶^Median [interquartile range], ^‡^Frequency (% within column), ^#^no statistic applicable on minimal observations.

^a^As applicable, respective statistics were done: ^b^Z score Mann–Whitney U-test for non-parametric data, ^c^Pearson’s chi-square test, respectively. ^d^A p-value >0.05 was considered statistically significant.

LOS: length of stay, ICU: intensive care unit, BMI: body mass index, HNFC: high-flow nasal cannula, NIV: non-invasive ventilation, IPPV: intermittent positive pressure ventilation, VTE: Venous thromboembolism, PE: pulmonary embolism, DVT: deep venous thrombosis, RRT: renal replacement therapy, SLED: sustained low-efficiency dialysis, CRRT: continuous renal replacement therapy, ECMO: extracorporeal membrane oxygenation, NVD: normal vaginal delivery, LSCS: lower segment caesarean section, CSE: combined spinal and epidural anesthesia, GA: general anesthesia.

## References

[1] CDC (2023). People with certain medical conditions. CDC.

[2] Englund JA, Chu HY (2018). Respiratory virus infection during pregnancy: Does it matter?. J Infect Dis.

[3] Mosby LG, Rasmussen SA, Jamieson DJ (2011). 2009 pandemic influenza A (H1N1) in pregnancy: a systematic literature review. Am J Obstet Gynecol.

[4] Siston AM, Rasmussen SA, Honein MA, Fry AM, Seib K, Callaghan WM (2010). Pandemic 2009 influenza A(H1N1) virus illness among pregnant women in the United States. JAMA.

[5] Cervantes-Gonzalez M, Launay O (2010). Pandemic influenza A (H1N1) in pregnant women: impact of early diagnosis and antiviral treatment. Expert Rev Anti Infect Ther.

[6] Shay M, MacKinnon AL, Metcalfe A, Giesbrecht G, Campbell T, Nerenberg K (2020). Depressed mood and anxiety as risk factors for hypertensive disorders of pregnancy: a systematic review and meta-analysis. Psychol Med.

[7] Kotlar B, Gerson E, Petrillo S, Langer A, Tiemeier H (2021). The impact of the COVID-19 pandemic on maternal and perinatal health: a scoping review. Reprod Health.

[8] Santa S, Doku DA, Olwal CO, Brown CA, Tagoe EA, Quaye O (2022). Paradox of COVID-19 in pregnancy: are pregnant women more protected against or at elevated risk of severe COVID-19?. Future Microbiol.

[9] Minisha F, Farrell T, Abuyaqoub S, Rahim AA, Ahmed H, Omer M (2022). Maternal risk factors of COVID-19-affected pregnancies: A comparative analysis of symptomatic and asymptomatic COVID-19 from the Q-PRECIOUS registry. Qatar Med J.

[10] Abdalla EOI, Nahid S, Valappil SS, Gudavalli S, Sellami S, Korichi N (2022;). Impact of COVID-19 status on patients receiving neuraxial analgesia during labor: A national retrospective-controlled study. Qatar Med J.

[11] Al-qassem AK, Humaidi AB, Al-kuwari AK, Hasan EM, Nosaiba H (2022;). Association between pregnancy and severe COVID-19 symptoms in Qatar: a cross-sectional study. medRxiv.

[12] Bachani S, Arora R, Dabral A, Marwah S, Anand P, Reddy KS (2021). Clinical profile, viral load, maternal-fetal outcomes of pregnancy with COVID-19: 4-week retrospective, tertiary care single-centre descriptive study. J Obstet Gynaecol Can.

[13] Liu D, Li L, Wu X, Zheng D, Wang J, Yang L (2020). Pregnancy and perinatal outcomes of women with coronavirus disease (COVID-19) pneumonia: a preliminary analysis. Am J Roentgenol.

[14] Massarotti C, Adriano M, Cagnacci A, Gorlero F, Gustavino C, Vallerino G (2021). Asymptomatic SARS-CoV-2 infections in pregnant patients in an Italian city during the complete lockdown. J Med Virol.

[15] Dيaz-Corvillَn P, Mِnckeberg M, Barros A, Illanes SE, Soldati A, Nien JK (2020). Routine screening for SARS CoV-2 in unselected pregnant women at delivery. PLoS One.

[16] Wang Y, Liang X, Wang H, Li L, Xiong G, Mi L (2020). A considerable asymptomatic proportion and thromboembolism risk of pregnant women with COVID-19 infection in Wuhan, China. J Perinat Med.

[17] Mattar CN, Kalimuddin S, Sadarangani SP, Tagore S, Thain S, Thoon KC (2020). Pregnancy Outcomes in COVID-19: A Prospective Cohort Study in Singapore. Ann Acad Med Singapore.

[18] Yang R, Mei H, Zheng T, Fu Q, Zhang Y, Buka S (2020). Pregnant women with COVID-19 and risk of adverse birth outcomes and maternal-fetal vertical transmission: a population-based cohort study in Wuhan, China. BMC Med.

[19] Allotey J, Stallings E, Bonet M, Yap M, Chatterjee S, Kew T (2020). Clinical manifestations, risk factors, and maternal and perinatal outcomes of coronavirus disease 2019 in pregnancy: living systematic review and meta-analysis. BMJ.

[20] Panagiotakopoulos L, Myers TR, Gee J, Lipkind HS, Kharbanda EO, Ryan DS (2020). SARS-CoV-2 infection among hospitalized pregnant women: reasons for admission and pregnancy characteristics - eight US. health care centers, March 1–May 30, 2020. Morb Mortal Wkly Rep.

[21] Vousden N, Bunch K, Morris E, Simpson N, Gale C, O’Brien P (2021). The incidence, characteristics and outcomes of pregnant women hospitalized with symptomatic and asymptomatic SARS-CoV-2 infection in the UK from March to September 2020: A national cohort study using the UK Obstetric Surveillance System (UKOSS). PLoS One.

[22] Jimenez RR, Garcell HG, Garcيa FG (2022). Retrospective study of risk factors for mortality in critically ill patients with COVID-19. J Emerg Med Trauma Acute Care.

